# Response to Smith et al. 18 February 2016

**DOI:** 10.1111/eva.12381

**Published:** 2016-05-17

**Authors:** Susan M. Haig

**Affiliations:** ^1^ U.S. Geological Survey, Forest and Rangeland Ecosystem Science CenterCorvallisORUSA

**Keywords:** conservation genetics, endangered species, endangered species act, wildlife management

In a recent *Evolutionary Applications* paper, Haig et al. (2016) presented an idea for the formation of a National Center for Small Population Biology. The paper was invited as part of a special issue dedicated to women in science (Wellenreuther and Otto 2016) where authors were asked to add a section to their papers that explored the idea of ‘what would you do now in your career if you could do anything’. The Haig et al. paper focused on my scientific contributions; hence, the ‘dream’ section discussed a potential means to address my career‐long interest in the science of small population conservation.

Upon revisiting the paper in light of Smith et al., I found that an earlier version of the Haig et al. manuscript was inadvertently submitted and subsequently published by the journal, resulting in an unintended and inaccurate focus on the U.S. Fish and Wildlife Service (USFWS). The published paper was not vetted in accordance with the U.S. Geological Survey (USGS) policies meant to ensure the quality, utility, and integrity of USGS science. To meet these policies, USGS requires that information products, including journal articles, by USGS scientists receive review and approval at the highest level of the agency which confers the full weight of agency endorsement.

Thus, the published version of Haig et al. does not represent the views of the USGS. I regret this error, particularly as USFWS is a close partner and we appreciate their scientific expertise.

The ‘dream’ section was not intended to be specifically directed at the USFWS, nor did we attempt to identify the range of agencies and organizations that use specialized scientific information to conserve small populations. Rather, it focused on improving the scientific information available to all conservation groups and addressed the needs of wild species conservation in many parts of the world—similar to the work carried out for captive populations by the IUCN Conservation Breeding Specialist Group (CBSG: www.cbsg.org). Thus, it was meant to provide a broad overview rather than propose the specific details required for a National Center for Small Population Biology. Our vision was to optimize integration and application of existing expertise (including that of USFWS, USGS, and other agencies and institutions), regardless of where it would reside institutionally, as well as to expand capacities to meet specialized information needs for conserving small populations.

The proposed Center would be multidisciplinary, drawing expertise from multiple fields (e.g., genetics, demography, ecology, decision science, policy, etc.), agencies, groups, and individuals to promote optimal endangered species recovery assessments. The genetic capabilities of USFWS described in Smith et al. (2016) are examples of the kinds of expertise needed in greater capacity.

I appreciate the support that Smith et al. (2016) offer for the overall concept of a National Center for Small Population Biology. They, like many of us, recognize a need for better development of and accessibility to endangered species information in the USA and abroad. Thus, I look forward to continuing our work with the many USFWS biologists with whom we have partnered for more than 30 years to share expertise and perceptions of what might provide for an enhanced model of endangered species research. I am optimistic that discussions regarding a potential National Center for Small Population Biology will continue as a means of recognizing the importance of the types, number, and scale of analyses that can be used to best assess species at risk.
